# Excretion of Urinary Metabolites of the Phthalate Esters DEP and DEHP in 16 Volunteers after Inhalation and Dermal Exposure

**DOI:** 10.3390/ijerph15112514

**Published:** 2018-11-09

**Authors:** Annette M. Krais, Christina Andersen, Axel C. Eriksson, Eskil Johnsson, Jörn Nielsen, Joakim Pagels, Anders Gudmundsson, Christian H. Lindh, Aneta Wierzbicka

**Affiliations:** 1 Division of Occupational and Environmental Medicine, Institute of Laboratory Medicine, Lund University, SE-22363 Lund, Sweden; jorn.nielsen@med.lu.se (J.N.); christian.lindh@med.lu.se (C.H.L.); 2 Division of Ergonomics and Aerosol Technology, Department of Design Sciences, Lund University, Box 118, SE-22100 Lund, Sweden; christina.andersen@design.lth.se (C.A.); axel.eriksson@design.lth.se (A.C.E.); joakim.pagels@design.lth.se (J.P.); anders.gudmundsson@design.lth.se (A.G.); aneta.wierzbicka@design.lth.se (A.W.); 3 Truly Labs AB, Medicon Village, SE-22381 Lund, Sweden; eskil@trulylabs.com

**Keywords:** phthalate esters, human exposure studies, human biomonitoring, indoor air pollution, indoor environment

## Abstract

Phthalate esters are suspected endocrine disruptors that are found in a wide range of applications. The aim of this study was to determine the excretion of urinary metabolites in 16 individuals after inhalation and/or dermal exposure to 100–300 µg/m^3^ of deuterium-labelled diethyl phthalate (D_4_-DEP) and bis(2-ethylhexyl) phthalate (D_4_-DEHP). Dermal exposure in this study represents a case with clean clothing acting as a barrier. After inhalation, D_4_-DEP and D_4_-DEHP metabolites were excreted rapidly, though inter-individual variation was high. D_4_-DEP excretion peaked 3.3 h (T½ of 2.1 h) after combined inhalation and dermal exposure, with total excreted metabolite levels ranging from 0.055 to 2.351 nmol/nmol/m^3^ (nmol of urinary metabolites per phthalates air concentration in (nmol/m^3^)). After dermal exposure to D_4_-DEP, metabolite excretion peaked 4.6 h (T½ of 2.7 h) after exposure, with excreted metabolite levels in between 0.017 and 0.223 nmol/nmol/m^3^. After combined inhalation and dermal exposure to D_4_-DEHP, the excretion of all five analysed metabolites peaked after 4.7 h on average (T½ of 4.8 h), and metabolite levels ranged from 0.072 to 1.105 nmol/nmol/m^3^ between participants. No dermal uptake of particle phase D_4_-DEHP was observed. In conclusion, the average excreted levels of metabolites after combined inhalation and dermal exposure to D_4_-DEP was three times higher than after combined exposure to D_4_-DEHP; and nine times higher than after dermal exposure of D_4_-DEP. This study was made possible due to the use of novel approaches, i.e., the use of labelled phthalate esters to avoid the background concentration, and innovative technique of phthalate generation, both in the particle and the gas phase.

## 1. Introduction

Outdoor pollution is well known to affect human health [1], while the impact of indoor environments on human health needs to be more intensively investigated. As we spend most of our time indoors, it is crucial to understand the health effects caused by pollutants present in indoor air. Indoor environments are not only polluted from outdoor sources, but also by indoor activities, such as cooking, cleaning, or the use of numerous consumer products, in addition to the presence of building materials [2,3].

Many indoor pollutants belong to the family of semivolatile organic compounds (SVOCs), being present both in the gas phase and as airborne particles [2]. Phthalate esters are SVOCs that are found in a wide range of applications, such as food packaging, building materials, personal care products, and children’s toys [4,5,6]. Since phthalate esters are not covalently bound to the product matrix, they can easily leak into the indoor environment [7], and they have been detected in air [8], dust [6], food, and water [9]. There has been recent interest in dermal absorption of phthalates directly from air [10,11], and some modelling approaches estimated the uptake from air-to-skin to be comparable to inhalation intake for some SVOCs [11,12].

Since 2015, the usage of the phthalate ester bis(2-ethylhexyl) phthalate (DEHP) has been restricted to 0.1% by weight in childcare articles and toys [13], and DEHP has been included into the REACH Candidate List as Substances of Very High Concern (SVHC) [14]. Several studies have linked phthalate esters with disturbance of the male reproductive system [15], asthma [16,17], allergic reactions [18,19], and even carcinogenesis, cardiotoxicity, hepatotoxicity, and nephrotoxicity [20,21,22].

Due to the widespread use of phthalate esters, humans are constantly exposed by multiple routes, such as ingestion [23], dermal exposure [24], or inhalation [4,25]. Phthalate metabolites have been detected in children’s urine (281 ng/mL, median value) [26], blood (41 ng/mL, median value) [27], and breast milk (143 ng/mL, median value) [28].

**Figure 1:**
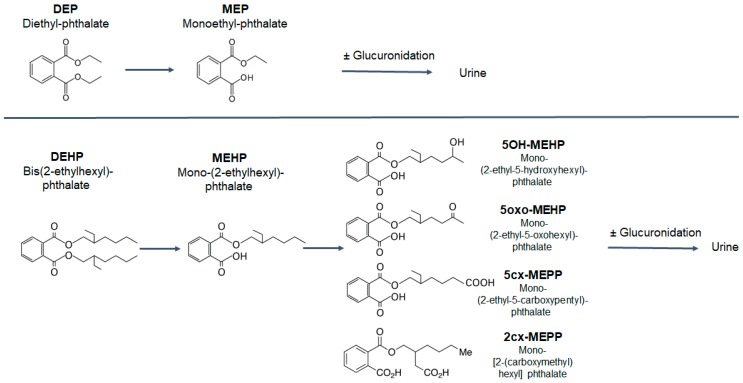
Analysed metabolites of diethyl phthalate (DEP) and bis(2-ethylhexyl) phthalate (DEHP) in this study, adapted from Koch et al. [23].

In the body, phthalate esters undergo rapid metabolism to the monoesters, and oxidized metabolites are excreted into the urine either freely or conjugated as glucuronides [29,30]. Figure 1 shows the most common metabolites of diethyl phthalate (DEP) and DEHP. The metabolites of the low-molecular-weight phthalate esters, such as DEP, usually undergo rapid deesterfication to the monoesters, which are excreted quickly [23]. Phthalate esters with five or more carbons in the ester side chain, such as DEHP, are first transformed to the monoester (e.g., MEHP), which can be further oxidized (Figure 1). Therefore, human exposure to the low-molecular-weight phthalates can be adequately assessed with urinary monoesters, but the oxidized metabolites need to be taken into account for exposure to the high-molecular-weight phthalates.

The aim of this human chamber exposure study was to investigate the uptake of two airborne phthalate esters, the volatile DEP and the less volatile DEHP, after inhalation and/or dermal exposure directly from air [31]. To our knowledge, this is the first human exposure study investigating the uptake of DEHP aerosol particles by inhalation and via the skin. Details on the technical set-up for the exposure, as well as particle generation, has just been published by Andersen et al. [31], while the work presented, here, describes the excretion of urinary metabolites. By analysing deuterium-labelled metabolites, we could avoid interference from exposure to phthalate esters from other sources. Knowledge of the uptake and excretion kinetics of these two phthalate esters is crucial to estimating our daily exposure as well as for correct risk assessment in humans.

## 2. Materials and Methods

### 2.1. Chemicals and Reagents

D_4_-labelled DEP and DEHP, as well as the internal standards ^13^C_6_-D_4_-labelled DEP and DEHP, D_4_-labelled MEP, MEHP, 5-OH-MEHP, 5-oxo-MEHP, 5-cx-MEPP, 2-cx-MMHP, and the internal standards D_9_-MEP and D_9_-5cx-MEPP, were all purchased from Toronto Research Chemicals, Toronto, ON, Canada.

### 2.2. Exposures and Set-Up

The study was approved by The Ethical Committee at Lund University (Dnr 2016/130) and was performed in accordance with the Declaration of Helsinki, including obtaining informed written consent from all subjects. The participants were all healthy and non-smokers, and none of the females were pregnant. Before exposure, all the participants underwent a medical examination, and all participants’ lung function values were classified as normal [31].

We exposed 16 individuals (9 females and 7 males) aged 19–47 using a 22 m^3^ stainless steel chamber. The participants were exposed separately to the two deuterium-labelled phthalate esters, D_4_-DEP and D_4_-DEHP, in four different exposure scenarios: (1) inhalation and dermal exposure to D_4_-DEP; (2) inhalation and dermal exposure to D_4_-DEHP; (3) dermal exposure to D_4_-DEP only; (4) dermal exposure to D_4_-DEHP only; see Andersen et al. [31]. For dermal exposures, the exposure to the two phthalate esters by inhalation was eliminated by having the participants breathe clean air through a hood that was sealed around the neck [31]. Air concentrations of phthalate esters in the particle phase were monitored by SMPS (scanning mobility particle sizer) (in-house construction [31]), HR-ToF-AMS (Aerodyne Inc., Billerica, MA, USA), and GC-MS/MS [31]. Each of the four exposure scenarios was carried out with four persons sitting in the chamber for three hours, with an at least seven days wash-out period between the different exposure scenarios. As we used deuterium-labelled phthalate esters for exposure, no instructions were given in this study to avoid phthalate-containing personal care products. However, participants had to directly shower before and after exposure with phthalate-free soap [31].

### 2.3. Collection of Airborne Phthalate Esters

Airborne DEHP (in both the gas phase and the particle phase) was collected using Teflon membrane particle filters (PTFE on Polypropylene Support, 37 mm, 0.45 µm, SKC, Eighty Four, PA, USA) followed by sorbent Tenax tubes, Tenax TA OVS (No. 226-56: glass fibre filter, Scantec Nordic, Sävedalen, Sweden). DEP was collected using only Tenax tubes. For each exposure, two triplicate sets, each lasting 1.5 h (in triplicate), of particle filters and Tenax tubes were sampled. Particle filters in Tenax tubes (particle phase) and Tenax layers (gas phase) were extracted individually with 1 mL of toluene containing 10 µL (1 µg) of internal standard, according to OSHA protocol No. PV2076 (Occupational Health and Safety Administration, USA Department of Labor, May 2001). Samples were sonicated for 30 min and centrifuged (10 min, 3000 rpm), and supernatants were transferred to HPLC glass vials (Agilent Technologies, Waldbronn, Germany).

### 2.4. GC-MS/MS Instrumentation and Conditions

The GC-MS/MS analysis method was adapted from Weiss et al. [32] and further modified. Analysis was performed on a Shimadzu GC 2010-Plus chromatograph (Shimadzu Corporation, Kyoto, Japan) coupled to a GC TQ8040 triple quadrupole mass spectrometer (Shimadzu Corporation). Extract (1 μL) was injected onto GC-MS/MS. The capillary column used was a HP-5MS-UI (15 m × 0.25 mm, 0.25 μm, Agilent Technologies, Waldbronn, Germany). Helium was the carrier gas at a flow rate of 1.5 mL/min, and argon was used as quenching gas at 2.25 mL/min. The temperature programme was initial temperature 60 °C for 1.5 min; ramp at 20 °C/min to 220 °C and held for 1 min; ramp at 5 °C/min to 280 °C and held for 4 min; injection at oven temperature of 280 °C; and transfer line of 280 °C. Sample injection volume was 1 μL, and a splitless injection mode was used. Electron impact ionisation was performed at 70 eV energy, and at a 280 °C ion source temperature. The quadrupole temperature was 280 °C. The MS was operated in selected reaction mode (SRM). The determined LOD ranged from 1.2 ng/mL (D_4_-DEP) to 5.5 (D_4_-DEHP) ng/mL. For more details see Supplementary Information (Table S5).

### 2.5. Urinary Metabolites—Sample Preparation

Urine samples were collected right before each exposure, right after exposure, and an hour after ended exposure. Participants were asked to collect all urine for 24 h at regular intervals, i.e., every hour. Urine was collected for maximal 24 h. Pilot studies performed in our lab showed that excreted levels of metabolites were below the detection limit already after 24 h after exposure. Urine was stored at −20 °C prior to analysis. Creatinine and urine density were measured, and 0.2 mL of supernatants was transferred to a glass insert (Teknolab Sorbent, Kungsbacka, Sweden) of a 96-well Rittner plate (Teknolab Sorbent). β-Glucuronidase was used to hydrolyse possible glucuronide conjugates of the metabolites. Therefore, 0.1 mL of ammonium acetate (pH 6.5) and 0.01 mL glucuronidase (*Escherichia coli*) were added, and the solution was incubated at 37 °C for 30 min [33]. After addition of 0.025 mL of a 50:50 (*v*/*v*) water/acetonitrile solution and 0.025 mL of D_9_-5cx-MEPP (internal standard), the plates were centrifuged for 10 min at 3000 rpm, prior to injection onto LC-MS/MS.

### 2.6. LC-MS/MS Instrumentation and Conditions

Samples were analysed for the five main D_4_-DEHP metabolites: D_4_-lablled mono-(2-ethyl-5-hydroxyhexyl) phthalate (5OH-MEHP), mono-(2-ethyl-5-oxohexyl)phthalate (5oxo-MEHP), mono-(2-ethyl-5-carboxypentyl)phthalate (5cx-MEPP) and mono-[2-(carboxymethyl), hexyl]phthalate (2cx-MMHP) [33]. Extract (1 μL) was injected onto LC-MS/MS. A C18 column (2.1 mm i.d. × 50 mm; Grace Genesis Lighting; Sunrise, FL, USA) was used before the injector to reduce the interference of contaminants during the mobile phase. A C18 column (1.5 μm, 2.0 mm i.d. × 30 mm VisionHT; Grace) was used, and the mobile phases were water and acetonitrile with 0.1% formic acid. The samples were analysed on a Shimadzu UFLC system (Shimadzu Corporation, Kyoto, Japan) coupled to a QTRAP5500 (triple quadrupole linear ion trap mass spectrometer) equipped with a TurboIon Spray source (AB Sciex, Framingham, MA, USA). The samples were analysed in duplicates. The determined LOD for all analysed metabolites were 0.5 ng/mL (D_4_-MEP), 0.9 ng/mL (D_4_-MEHP), 0.1 ng/mL (D_4_-5OH-MEHP), 0.2 ng/mL (D_4_-5oxo-MEHP), 0.1 ng/mL (D_4_-5cx-MEPP), and 0.1 ng/mL (D_4_-2cx-MMHP). For more details, see Supplementary Information (Table S6).

### 2.7. Calculation of Total Excreted Dose

The total dose of phthalate ester available in the lungs was estimated individually for each participant. With a tidal volume of 6 mL/kg bodyweight [34] and a breathing rate of 12 breaths/min [35], respiratory minute volumes were calculated, and these were between 4.32 and 6.26 L/min for women and between 4.90 and 9.50 L/min for men. These are lower in comparison to respiratory minute volumes reported by the EPA exposure handbook that range for people in a sedentary state, from 6.5 L/min (age 30–60) to 6.7 L/min (age 18–30) in women, and from 8.3 L/min (age 30–60) to 8.7 L/min (age 18–30) in men [35]. The individual respiratory minute volumes (in L/min) was then multiplied by 180 min (length of exposure), and the air concentration of phthalate ester, in order to estimate a total lung dose of phthalate esters (in nmol). Particle deposition in the airways is dependent on size distribution and breathing frequency. It is, therefore, not correct to assume that 100% of the inhaled particles deposit in the airways [31]. The deposited fraction of DEHP particles in the lungs after inhalation exposure was calculated using the MPPD model with the specific size distributions that were measured during the exposures, resulting in 26% of particle deposition of inhaled DEHP; see Andersen et al. [31].

### 2.8. Pharmacokinetic Evaluation

Pharmacokinetic evaluation was performed using Phoenix WinNonlin version 8 (Certara, Princeton, NJ, USA). Non-compartmental analysis was performed using the following settings: uniform weighting, plasma (200–202) model type, linear up log down calculation, dosing defined (extravascular for dermal only and IV infusion (3 h) for dermal and inhalation).

## 3. Results and Discussion

### 3.1. Metabolites in Urine after Inhalation and Dermal Exposure to D_4_-DEP

D_4_-DEP is metabolised in the body to the monoester D_4_-MEP (Figure 1). The excretion characteristics of D_4_-MEP were determined by analysing all urine samples from 16 volunteers after combined inhalation and dermal exposure, or by dermal exposure only.

**Figure 2:**
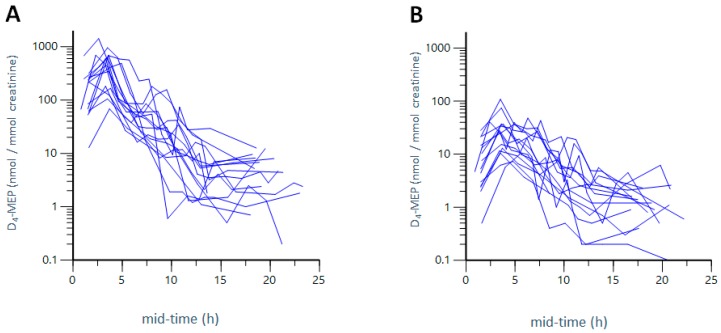
Excretion kinetics of D_4_-MEP. Excretion of the urinary metabolite D_4_-MEP from 16 participants over mid-time (mid time point of collection interval) after combined inhalation and dermal exposure to D_4_-DEP (**A**) and dermal only exposure to D_4_-DEP (**B**). The y-axis is presented in a logarithmic scale, and time points are given in mid-time (mid time point of collection interval).

Figure 2A,B show the excretion curves of D_4_-MEP after combined inhalation and dermal, exposure (Figure 2A), as well as after dermal exposure only (Figure 2B) to 1154–1759 nmol/m^3^ D_4_-DEP (~300 µg/m^3^). Figure 2A,B demonstrate that D_4_-MEP was eliminated quickly in both exposure scenarios and, 24 h after exposure, no metabolites could be detected. The metabolite was excreted in higher levels and slightly faster after the combined inhalation and dermal exposure (Figure 2A), than after dermal exposure only (Figure 2B). Table 1 presents the excretion characteristics as averages for all participants after three hours of exposure, including the time points (T_max_) of the maximum concentration (C_max_) and the excretion half-life (T½) of D_4_-MEP. The total excreted amount of metabolites is expressed as an area under the excretion curve (AUC) in the units nmol·h/mmol creatinine. Excretion characteristics for all individuals after three hours of exposure are presented in Table S1.

For the combined inhalation and dermal, exposure to D_4_-DEP, T_max_ for D_4_-MEP was calculated as 3.3 h and T½ as 2.1 h (average over all participants). The maximum metabolite concentration (C_max_) was measured as 513 nmol/mmol creatinine of excreted metabolite in urine per mmol creatinine (AUC of 1642 nmol·h/mmol creatinine) (Table 1).

Dermal uptake only was assessed by participants wearing hoods that supplied fresh air [31], as described in the method section. Participants wore loosely fitting hospital cotton clothes, leaving hands free for exposure, thereby creating a realistic exposure scenario with clothing acting as a barrier. After the dermal exposure, D_4_-MEP was excreted, on average, at T_max_ of 4.6 h, with a T½ of 2.7 h (Table 1). Excretion times were thus marginally longer after dermal exposure only, than after combined inhalation and dermal exposure (T_max_ 3.3 h). The excretion half-life (T½) was similar in both exposure scenarios. After dermal exposure to D_4_-DEP, C_max_ was calculated as an average of 34 nmol/mmol creatinine (AUC of 157 nmol·h/mmol creatinine), which is 15-fold (C_max_) or 10-fold (AUC) lower compared to the combined inhalation and dermal exposure of D_4_-DEP. The C_max_ and AUC for the inhalation only exposure was achieved by subtracting the values for C_max_ and AUC for the dermal exposure from the values obtained after the combined inhalation and dermal exposure. C_max_ for inhalation exposure was calculated as 446 nmol/mmol creatinine, with a total AUC of 1342 nmol·h/mmol creatinine (average of all participants), which accounts for 82% (C_max_) and 87% (AUC) of the combined exposure (inhalation and dermal).

Table 1 and Table S1 demonstrate that the excretion characteristics differed greatly between the individuals. For the combined inhalation and dermal exposure to D_4_-DEP, C_max_ ranged from 68.4 to 1423 nmol/mmol creatinine, with a coefficient of variation (CV) of 71% reflecting inter-individual differences while, after dermal exposure to D_4_-DEP, excreted levels ranged from 7.30 to 109 nmol/mmol creatinine (CV = 81%). Values for excreted metabolites after inhalation only were achieved by subtracting levels after dermal exposure only from the levels after the combined inhalation and dermal exposure. This resulted in total excreted metabolite levels ranging from 48.1 to 1375 nmol/mmol creatinine after inhalation exposure to D_4_-DEP only. The inter-individual variety makes it difficult to calculate significant differences. However, on average over all participants, combined inhalation and dermal exposure accounted for 15-fold higher metabolite levels (C_max_) compared to dermal exposure only. Calculated C_max_ levels after inhalation exposure only were 13-fold higher than levels after dermal exposure only.

### 3.2. Metabolites in Urine after Inhalation and Dermal Exposure to D_4_-DEHP

D_4_-DEHP is transformed in the body into five main metabolites (Figure 1). Their excretion characteristics were determined by analysing all urine samples from 16 volunteers after inhalation and dermal exposure, or by dermal exposure only.

**Figure 3:**
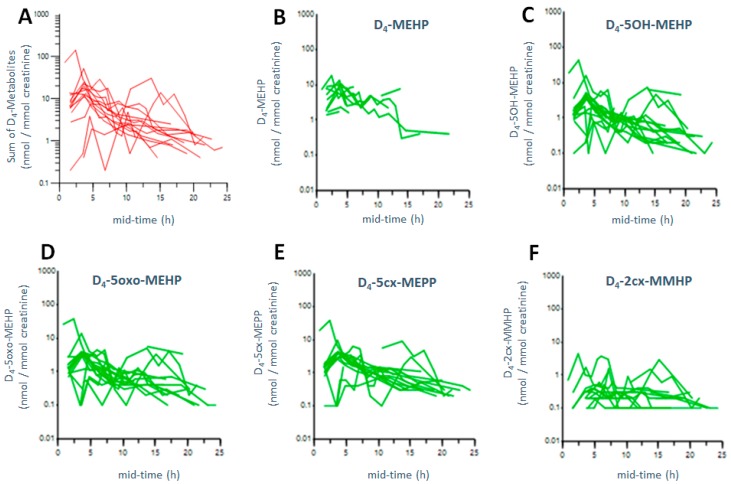
Excretion kinetics of D_4_-DEHP metabolites. Urinary metabolites from 16 participants over mid-time (mid time point of collection interval) after combined inhalation and dermal exposure of D_4_-DEHP: sum of all five analysed metabolites (**A**), and D_4_-MEHP (**B**), D_4_-5OH-MEHP (**C**), D_4_-5oxo-MEHP (**D**), D_4_-5cx-MEPP (E), D_4_-2cx-MMHP (**F**), respectively. The y-axis is presented on a logarithmic scale, and time points are given in mid-time (mid time point of collection interval).

Metabolite levels were below detection limit after dermal exposure of D_4_-DEHP. Figure 3A–F and Table 1 show that, after the combined inhalation and dermal exposure of D_4_-DEHP, metabolites were excreted rapidly, and 24 h after exposure, no urinary metabolites could be detected.

Figure 3 presents the excretion curves of the five urinary metabolites after three hours of combined inhalation and dermal exposure of 223–367 (nmol/m^3^) D_4_-DEHP (~100 D_4_-DEHP µg/m^3^). Detailed excretion characteristics are described in Table S2, including C_max_, T_max_, and T½ of all five analysed metabolites after the combined inhalation and dermal exposure to D_4_-DEHP. Of the five metabolites analysed, D_4_-MEHP was excreted most rapidly at 4.1 h (T_max_), and at the highest C_max_ levels, with 8.2 nmol/mmol creatinine (average over all participants). The secondary metabolites D_4_-5OH-MEHP, D_4_-5oxo-MEHP, and D_4_-5cx-MEPP were excreted after 4.9 h–5.2 h (T_max_), with an average C_max_ of 7.3 nmol/mmol creatinine (D_4_-5OH-MEHP), 6.3 nmol/mmol creatinine (D_4_-5oxo-MEHP), and 3.1 nmol/mmol creatinine (D_4_-5cx-MEPP). The last metabolite excreted at 6.4 h (T_max_) was D_4_-2cx-MMHP at average C_max_ levels of only 0.8 nmol/mmol creatinine.

Average levels over all participants are presented in Table 1. The C_max_ of the sum of all five metabolites was excreted on average, after a T_max_ of 4.7 h, with a T½ of 4.8 h. T_max_, as well as T½, was thus longer compared to combined exposure of D_4_-DEP (T_max_ 3.3 h; T½ = 2.1 h). C_max_ of all metabolites was calculated, on average, as 27.4 nmol/mmol creatinine (AUC of 121 nmol·h/mmol creatinine). Compared to the combined exposure of D_4_-DEP, exposure of D_4_-DEHP led to 19-fold lower C_max_ levels and 14-times lower AUC. The delayed excretion, as well as lower levels of metabolites, could be explained by a delayed uptake of the particle phase D_4_-DEHP compared to the uptake of the gas phase D_4_-DEP. However, direct comparison of the excreted dose between DEHP and DEP are difficult, due to the different levels of phthalate concentrations in the air. While DEP could be generated at levels of 300 µg/m^3^, DEHP aerosols could be only generated in concentrations of 100 µg/m^3^; see Andersen et al. [31].

To our knowledge, no human exposure study on inhalation or dermal exposure to airborne particle phase DEHP has been performed. Other human biomonitoring studies on oral intake of DEHP measured T½ for urinary DEHP metabolites as 4–8 h [36] and 2–10 h [23], depending on the metabolite. Half-lives reported in our study were in a similar range (T½ 4.7 h). In our study (see above), T_max_ of the five DEHP metabolites were found at 4.1 h (MEHP), 4.9–5.2 h (D_4_-5OH-MEHP, D_4_-5oxo-MEHP and D_4_-5cx-MEPP), and 6.4 h (D_4_-5cx-MHHP). Koch et al. reported similar values for T_max_ as 2 h (MEHP), 4 h (5OH-MEHP, 5cx-MEPP and 5oxo-MEHP), and 8–10 h (2cx-MMHP) [23]. Compared to the values reported by Koch et al. [23], T_max_ was, in our study, similar for most compounds, except MEHP (longer in our study) and 2cx-MMHP (marginally shorter in our study). This might be due to a slower uptake from the intestine after oral exposure, or individual variation, as the study from Koch et al. presents data from one person only [23].

Furthermore, oral exposure of DEHP, described by Koch et al., led to excretion of the metabolites in two different phases, after absorption and distribution [23], which could not be detected in our study. No second excretion phase was detected for the DEHP metabolites in our study, which could be explained by differences in metabolism after inhalation versus oral uptake. Possibly, two-phase excretion could not be monitored, because levels of urinary metabolites for some individuals in our experiments were very close to the detection limit (0.1–0.9 ng/mL, see Supplementary Information). In our study, oxidised metabolites were excreted at equally high urinary levels compared to the monoester, but with slightly delayed excretion times. Thus, these secondary metabolites, namely 5-OH-MEHP, 5-oxo-MEHP, and 5cx-MEPP, could serve as good exposure biomarkers for human exposure to phthalate esters, which has been already suggested in earlier studies [23,36]. Furthermore, these oxidized metabolites are more specific biomarkers than their monoesters, as phthalate monoesters are considered environmental contaminants on their own [32].

### 3.3. Total Excretion in Relation to Estimated Uptake: Inhalation of DEP vs. DEHP

Due to the presence of D_4_-DEHP in the particle phase, deposition of particles in the respiratory tract should be taken into account. DEHP particles that deposit in the alveolar region might enter the bloodstream, while particles that deposit in the tracheobronchial and extrathoracic regions might be moved up to the respiratory tract, swallowed, and ingested via the stomach, where they could undergo a similar metabolism as an oral dose of phthalates [31].

The deposited (and thus bioavailable) fraction of the inhaled D_4_-DEHP particles was calculated as 26% on a mass basis; for further details, see Andersen et al. [31]. We normalised the total (cumulative) amount of urinary metabolites (in nmol) to the estimated total dose of phthalate esters available in the lung during the three hours of exposure (Table S3). The total amount of phthalate esters in the lung was estimated using the individual breathing volume, and the air concentration of the phthalate ester of each individual’s exposure (see Materials and Methods). Results are given in total amounts of metabolites (in nmol) per total available dose of D_4_-DEHP in the lung (in nmol).

As no uptake could be detected after dermal exposure to D_4_-DEHP, we assumed that uptake of combined inhalation and dermal exposure to D_4_-DEHP consisted of uptake by inhalation only. Inhalation of D_4_-DEHP resulted in an average excretion of 81 nmol of metabolites (sum of all five metabolites). Normalisation to the total bioavailable dose in the lung led to average levels of 0.995 nmol/nmol available phthalate ester in the lung, suggesting that individuals excreted 99.5% of the estimated D_4_-DEHP lung dose (Table S3).

For comparison, we also estimated the total bioavailable amount in the lungs after inhalation only of D_4_-DEP. Therefore, we subtracted the amount of metabolites after dermal exposure form the amount of metabolites found after the combined dermal and inhalation exposure (Table S3). Assuming 100% of D_4_-DEP to be available in the lungs due to its presence in the gas phase [4], we calculated average excretion of 84% (0.840 nmol/nmol lung dose) of the estimated dose of D_4_-DEP. Thus, the excreted dose after inhalation of D_4_-DEP is slightly lower than after inhalation of D_4_-DEHP (84% versus 99.5%, respectively) once the bioavailable dose in the lung is taken into account. Andersen et al. [31] calculated the excreted dose of DEHP in this experiment to slightly lower levels of only 69%, using estimated breathing volumes of 7.5 L/min as an average of all participants [37]. In this study however, breathing volumes were calculated individually, according to the individual’s bodyweight [34]. There is no unified approach among scientists regarding the use of breathing volumes in different studies. While Davis et al. [34] offers an individual approach based on bodyweight, others consider these values to be underestimated, as they are based on mechanical respirator flows which have their own restrictions when applied to healthy individuals. This is an area where a unified approach would benefit further studies.

While the metabolism of DEHP is different after inhalation and ingestion [4], metabolites will—independently of their uptake mechanism—be mainly excreted via urine, and to 10% via faeces [38]. There are—to our knowledge—no human exposure studies on inhalation of airborne DEHP. Human biomonitoring studies on oral intake of DEHP reported that, up to 24 h after dosing of D_4_-DEHP, 67% of the dose was excreted as one of the five D_4_-DEHP metabolites determined in urine [23]. However, this study by Koch et al. was limited to one person, and another study by Anderson et al. with 20 test subjects reported an average of 45% of the dose being excreted 24 h after oral exposure to DEHP [36]. In our study, the excreted dose after inhalation of D_4_-DEHP was higher, ranging from 69% (calculated with average breathing rates of 7.5 L/min) [31] to 99.5% (calculated with individual breathing rates).

### 3.4. Total Urinary Excreted Dose: Inhalation versus Dermal Exposure

**Figure 4:**
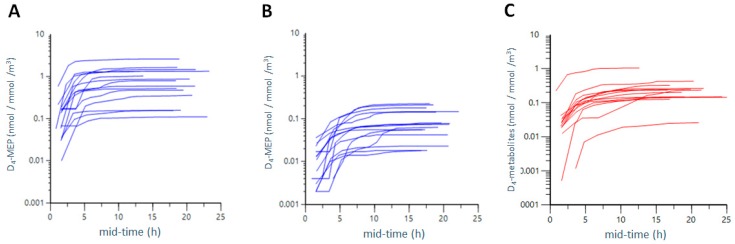
Total excreted dose after combined inhalation and dermal exposure to D_4_-DEP (**A**) and dermal exposure to D_4_-DEP only (**B**) as well as inhalation and dermal exposure of D_4_-DEHP (**C**). Cumulative excreted metabolite levels (in nmol) were normalised to air concentration of phthalate esters (in nmol/m^3^ air volume). The y-axis is presented on a logarithmic scale, and time points are given in mid-time (mid time point of collection interval).

To form a basis for comparing the different exposure scenarios, we calculated the total excreted urinary dose, expressed as the total (cumulative) amount of urinary metabolites (in nmol) normalised to the air concentration (nmol/m^3^) of phthalate ester (Figure 4A,B, Tables S3 and S4).

The combined inhalation and dermal exposure of D_4_-DEP led to a total excreted urinary amount of 1243 nmol D_4_-MEP (average of all participants). Normalized to air concentrations, this reflects an average excreted dose of 0.890 nmol metabolites per nmol/m^3^ (Table S4). Dermal exposure only led to lower levels of 119 nmol of excreted D_4_-MEP, which results in an average excreted dose of 0.094 nmol/nmol/m^3^ after normalization to air concentrations. Excreted metabolite levels, for inhalation exposure only, were obtained by subtracting excreted levels of D_4_-MEP after dermal exposure from excreted D_4_-MEP levels after combined inhalation and dermal exposure. Inhalation of D_4_-DEP thus resulted in an excreted dose of 0.790 nmol/nmol/m^3^.

Our data demonstrated that excretion of D_4_-MEP was, on average, nine times higher after combined inhalation and dermal exposure than after dermal only exposure to D_4_-DEP. Comparing only inhalation exposure to dermal exposure, the average excretion of D_4_-DEP after inhalation only was eight times higher than after dermal exposure. However, inter-individual variety of the excreted dose was wide, with values of CV between 74% and 83% (Table S4).

To calculate the inhalation exposure of D_4_-DEHP, the total excreted amount of all five measured urinary metabolites was added together, and this cumulative amount of metabolites (in nmol) was normalized to the air concentration of D_4_-DEHP (Figure 4C and Table S3). On average, 317 nmol was excreted as metabolites, which equates to an average excreted dose of 0.266 nmol/nmol/m^3^, and levels were thus three times lower compared to inhalation of D_4_-DEP (0.790 nmol/nmol/m^3^), as well as combined exposure to D_4_-DEP (0.890 nmol/nmol/m^3^). 

Uptake via inhalation or via the dermal pathways follows different mechanisms. Uptake by inhalation depends largely on the concentration, on the inhaled (and exhaled) volume, and on diffusion and solubility in the tissue, while dermal uptake depends on the deposition of the molecules on the skin, the lipid composition of the skin, the diffusion through the boundary layer of the skin and transport from the skin surface to the blood [10].

DEHP has been shown to barely penetrate human skin when applied purely and directly [39], but not much is known about dermal uptake from airborne particle phase concentrations of DEHP. The higher uptake of D_4_-DEP by combined exposure compared to dermal exposure only was expected, as clothing was acting as a barrier. In our study, only hands were exposed to airborne DEHP during skin exposure, and the observed differences might, therefore, be partly explained by the clothing acting as a barrier for air-to-skin transfer.

Higher levels of urinary metabolites have been reported earlier, when DEP was applied directly on the skin [40,41,42], suggesting increased exposure after direct topical application to the skin, compared to dermal absorption from airborne phthalates. In an exposure study with 26 participants, Janjua and coworkers showed that direct topical application of a cream containing 2% of DEP resulted in 5.79% of DEP being excreted, with a T_max_ of 8–12 h [42]. Only recently, dermal absorption from airborne compounds has been investigated [4,41,43]. Weschler et al. performed experiments with bare-skin participants who were exposed to DEP via inhalation and skin, with similar air concentrations compared to our study (300 µg/m^3^), and similar excretion times of MEP (4–8 h) [40]. Equal levels of excreted metabolites were found by Weschler et al. after inhalation (3.8 µg/µg/m^3^) and dermal exposure (4.0 µg/µg/m^3^), showing the strong influence of the amount of bare skin exposed [40]. In addition, Morrison et al. demonstrated that previously exposed clothing might play a crucial role in dermal uptake, showing higher uptake of DEP in participants wearing pre-exposed cotton clothing than bare-skin participants [41].

Our study group (*n* = 16) included volunteers aged 19–47, both women (*n* = 9) and men (*n* = 7). Due to the small number of participants, it is not possible to draw firm conclusions concerning age and gender. The observed differences in the excreted dose might not only depend on different breathing rates, but could possibly be explained by gender-specific differences in metabolic enzyme activities, or differences in lung physiology allowing faster permeability in the lungs. Phthalate metabolism might depend on different factors, such as age or sex [44]. Influence of age or gender on phthalate metabolism has been described by Koch et al., detecting higher levels of oxidised metabolites in children than in adults [45]. Gender-specific changes in girls and boys have been reported regarding phthalate metabolites and thyroid hormones [46], and markers of peripubertal metabolic homeostasis [47], respectively. Slight differences were observed in our study between women and men, however, these were not statistically significant and, therefore, are not discussed further.

## 4. Conclusions

This study has demonstrated that human participants excreted higher levels of urinary metabolites following combined inhalation and dermal exposure, compared to only dermal exposure, to airborne D_4_-DEP and D_4_-DEHP. The average excreted levels of metabolites after the combined inhalation and dermal exposure to D_4_-DEP were three times higher than after the combined exposure to D_4_-DEHP, and nine times higher than after only the dermal exposure to D_4_-DEP. No metabolites could be detected after the dermal exposure to airborne D_4_-DEHP.

In this study, we determined individual differences in metabolite excretion in a group of 16 volunteers during exposures to gas and particle phase phthalates, dermal exposure with clean clothes acting as a barrier, and in combined inhalation and dermal exposures. This was possible due to the use of novel approaches, i.e., use of labelled phthalate esters to avoid the background concentration, as well as innovative technique of phthalate generation, both in the particle and the gas phase.

## Figures and Tables

**Table 1:** Average urinary metabolites after three hours of inhalation and/or dermal exposure to D_4_-DEP and D_4_-DEHP, including the time points (T_max_) of the maximum concentration (C_max_) and the excretion half-life (T½) of the excreted metabolites, and AUC (area under the excretion curve). Presented values are averages, min and max values in brackets, and coefficient of coefficient of variation (CV).

Exposure		T_max_ (h) (Min–Max)	T½ (h) (Min–Max)	C_max_ (nmol/mmol Creatinine) (Min–Max)	AUC (nmol·h/mmol Creatinine) (Min–Max)
D_4_-DEP	Dermal exposure	4.6 (3.4–10) CV = 42%	2.7 (0.7–11) CV = 105%	34 (7.30 to 109) CV = 81%	157 (35.5–356) CV = 67%
	Inhalation and dermal exposure	3.3 (1.6–4.9) CV = 22%	2.1 (0.9–4.2) CV = 54%	513 (68.4 to 1423) CV = 71%	1642 (257–3437) CV = 63%
D_4_-DEHP	Dermal exposure	n.d.	n.d.	n.d.	n.d.
	Inhalation and dermal exposure	4.7 (1.5–14) CV = 69%	4.8 (0.6–11) CV = 73%	27.4 (2.5–141) CV = 126%	121 (43.4–340) CV = 72%

## Supplementary Materials

The following are available online at http://www.mdpi.com/1660-4601/15/11/2514/s1. Table S1: Excretion kinetics of D_4_-DEP metabolites, Table S2: Excretion kinetics of D_4_-DEHP metabolites, Table S3: Total excreted dose after inhalation exposure to D4-DEP and D4-DEHP, Table S4: Total excreted dose after combined inhalation and dermal and dermal exposure to D4-DEP, Table S5: Analytical details for quantification of phthalate esters using GC-MS/MS, Table S6: Analytical details for quantification of phthalate metabolites using LC-MS/MS.

## Abbreviations

DEP	diethyl phthalate
MEP	monoethyl phthalate
DEHP	bis(2-ethylhexyl) phthalate
MEHP	mono(2-ethylhexyl)phthalate
5OH-MEHP	mono-(2-ethyl-5-hydroxyhexyl) phthalate
5oxo-MEHP	mono-(2-ethyl-5-oxo-hexyl) phthalate
5cx-MEPP	mono-(2-ethyl-5-carboxypentyl) phthalate
-cx-MMHP	mono-[2-(carboxymethyl) hexyl] phthalate
SMPS	scanning mobility particle sizer
HR-ToF-AMS	high-resolution time-of-flight aerosol mass spectrometer
GC-MS/MS	gas chromatography tandem mass spectrometry
LC-MS/MS	liquid chromatography tandem mass spectrometry
T_max_	excretion time
C_max_	the maximum concentration
T½	excretion half-life
AUC	area under the excretion curve
LOD	limit of detection
LOQ	limit of quantification
