# Supplementary material for: Excretion of Urinary Metabolites of the Phthalate Esters DEP and DEHP in 16 Volunteers after Inhalation and Dermal Exposure
Source: Int J Environ Res Public Health. 2018 Nov 9;15(11):2514. doi: 10.3390/ijerph15112514 (PMC6266104; doi:10.3390/ijerph15112514)
Supplement: Supplementary file 1 [file ijerph-15-02514-s001.pdf]

Supplementary Information

Table S1. Excretion kinetics of D_4-DEP metabolites.

Excretion characteristics of the urinary metabolite D_4-MEP from 16 participants after exposure to D_4-DEP by combined, inhalation and dermal, exposure and dermal exposure only. Characteristics include C_{max} (maximum concentration in nmol), T_{max} (time of maximum concentration), T½ (excretion half-life), and AUC (area under the excretion curve). Concentrations are presented in nmol / mmol creatinine (cr), T_{max} and T½ are given in mid-time (mid time point of collection interval).

DEP	Inhalation and dermal					Dermal					Inhalation only ((inhalation and dermal)-dermal)	
Subject (gender)	air concentration	T_{max}	T½	C_{max}	AUC	air concentration	T_{max}	T½	C_{max}	AUC	C_{max}	AUC
	nmol/m³	h	h	nmol/mmol cr	nmol·h /mmol cr	nmol/m³	h	h	nmol/mmol cr	nmol·h /mmol cr	nmol /mmol cr	nmol·h /mmol cr
**1 (F)**	1158	3.5	2.3	625	1828	1224	10.3	2.1	20.6	118	605	1710
**2 (F)**	1158	3.3	3.1	604	1396	1224	3.4	11.3	28.0	135	576	1262
**3 (F)**	1158	2.6	0.8	1423	3437	1224	6.3	1.8	48.0	245	1375	3192
**4 (F)**	1158	2.3	4.2	694	1821	1224	3.6	5.9	74.5	344	619	1477
**5 (F)**	1759	3.3	0.9	444	1397	1242	3.5	1.3	109	356	336	1041
**6 (M)**	1759	3.3	2.5	182	493.4	1242	3.5	2.1	10.5	44.6	172	449.8
**7 (F)**	1759	3.5	2.7	105	491.8	1242	3.7	2.5	13.0	60.5	92.2	431.4
**8 (M)**	1759	3.6	1.1	695	1999	1242	3.6	1.7	15.3	104	679	1895
**9 (M)**	1702	3.6	3.9	681	3385	1154	4.9	2.9	36.7	218	644	3167
**10 (F)**	n.a.	n.a.	n.a.	n.a.	n.a.	1154	3.4	0.9	25.9	98.7	n.a.	n.a.
**11 (F)**	1702	3.6	1.6	343	1370	1154	4.8	1.6	7.30	35.5	336	1334
**12 (M)**	1702	3.5	3.4	958	3269	1154	n.a.	n.a.	n.a.	n.a.	n.a.	n.a.
**13 (M)**	1268	3.8	0.9	166	732.8	1154	3.5	0.7	33.9	158	133	575
**14 (M)**	1268	4.9	1.1	489	1820	n.a.	n.a.	n.a.	n.a.	n.a.	n.a.	n.a.
**15 (F)**	1268	1.6	1.3	218	933.2	1631	3.8	1.0	36.9	216	181	716.8
**16 (M)**	1268	3.7	2.1	68.4	257.2	1631	6.2	1.6	20.3	59.3	48.1	197.8
**Mean**	**1456**	**3.3**	**2.1**	**513**	**1642**	**1260**	**4.6**	**2.7**	**34.3**	**157**	**446**	**1342**
**SD**	273.1	0.7	1.1	364	1043	155.4	1.9	2.8	27.7	105	363	967.2
**CV(%)**	19	22	54	71	63	12	42	105	81	67	81	72

Table S2. Excretion kinetics of D_4-DEHP metabolites

Excretion characteristics of the five urinary metabolites D_4-MEHP, D_4-5OH-MEHP, D_4-5oxo-MEHP, D_4-5cx-MEPP and D_4-2cx-MMHP from 16 participants after exposure to D_4-DEHP by inhalation exposure. Characteristics include C_{max} (maximum concentration in nmol), T_{max} (time of maximum concentration), T½ (excretion half-life), and AUC (area under the excretion curve). Concentrations are presented in nmol / mmol creatinine (cr), T_{max} and T½ are given in mid-time (mid time point of collection interval)

DEHP Inhalation															
**Subject (gender)**	**air conc.**	**all metabolites**				**D_4-MEHP**		**D_4-5OH-MEPP**		**D_4-5oxo-MEPP**		**D_4-5cx-MEPP**		**D_4-2cx-MMHP**	
		**T_{max}**	**T½**	**C_{max}**	**AUC**	**T_{max}**	**C_{max}**	**T_{max}**	**C_{max}**	**T_{max}**	**C_{max}**	**T_{max}**	**C_{max}**	**T_{max}**	**C_{max}**
	**nmol/ m³**	**h**	**h**	**nmol /mmol cr**	**nmol·h /mmol cr**	**nmol**	**nmol /mmol cr**	**nmol**	**nmol /mmol cr**	**nmol**	**nmol /mmol cr**	**nmol**	**nmol /mmol cr**	**nmol**	**nmol /mmol cr**
**1 (F)**	319	1.5	11	13.5	98.5	1.5	7.8	3.5	1.4	5.7	3.2	5.7	3.4	5.7	0.6
**2 (F)**	319	3.4	2.2	17.5	120	3.4	5.8	3.4	3.9	3.4	3.5	3.4	5.0	4.5	0.4
**3 (F)**	319	2.4	9.1	141	370	2.4	18	2.4	43	2.4	37	2.4	38	2.4	4.4
**4 (F)**	319	n.d.	n.d.	n.d.	n.d.	n.d.	n.d.	n.d.	n.d.	n.d.	n.d.	n.d.	n.d.	n.d.	n.d.
**5 (F)**	327	3.6	1.5	51.4	210	3.6	11	3.6	16	3.6	14	3.6	9.6	3.6	1.7
**6 (M)**	327	10	3.0	2.50	22.2	n.d.	n.d.	10	1.0	10	0.7	10	0.7	10	0.1
**7 (F)**	367	14	1.1	30.4	173	14	7.6	14	7.3	14	5.0	14	9.0	14	1.5
**8 (M)**	367	n.d.	n.d.	n.d.	n.d.	n.d.	n.d.	n.d.	n.d.	n.d.	n.d.	n.d.	n.d.	n.d.	n.d.
**9 (M)**	223	3.7	1.5	19.7	72.8	3.7	7.7	3.7	4.5	3.7	3.4	3.7	4.0	3.7	0.2
**10 (F)**	223	3.6	5.6	12.6	57.6	3.6	1.7	3.6	4.2	3.6	3.9	3.6	2.6	3.6	0.2
**11 (F)**	223	4.6	4.6	7.40	43.4	n.d.	n.d.	4.6	2.6	4.6	2.6	3.8	3.1	6.1	0.2
**12 (M)**	223	3.9	4.7	13.3	70.9	3.9	2.3	3.9	3.8	3.9	3.5	3.9	3.5	8.4	0.3
**13 (M)**	324	3.7	5.6	11.0	86.7	1.6	3.6	3.7	3.4	3.7	3.5	3.7	4.0	8.7	0.3
**14 (M)**	324	3.7	0.6	24.9	130	3.7	13	3.7	4.0	3.7	2.9	3.7	4.4	4.9	0.4
**15 (F)**	324	3.6	10	22.2	119	3.6	8.9	3.6	5.1	3.6	3.9	3.6	3.9	7.8	0.6
**16 (M)**	367	4.1	5.6	16.5	116	4.1	10.2	6.4	2.0	6.4	1.3	4.1	3.1	6.4	0.5
**Mean**	**306**	**4.7**	**4.8**	**27.4**	**121**	**4.1**	**8.2**	**5.0**	**7.3**	**5.2**	**6.3**	**4.9**	**3.1**	**6.4**	**0.8**
**SD**	52.6	3.2	3.5	34.6	87.3	3.2	4.7	3.1	11	3.1	9.4	3.1	1.2	3.1	1.1
**CV(%)**	17	69	73	126	72	78	57	63	149	60	149	63	38	48	140

Table S3. Total excreted dose after inhalation exposure to D_4-DEP and D_4-DEHP.
Excreted metabolite levels (in nmol) after inhalation given as cumulative amounts (nmol), as well as cumulative amounts normalized per total lung dose or air concentration. Lung available dose for D_4-DEP was estimated to 100% and for D_4-DEHP to 26 %.

		**DEP inhalation only**						**DEHP inhalation only**					
**Subject (gender)**	**weight (kg)**	**air conc.**	**total lung dose**	**available lung dose (100%)**	**cumulative amount metabolites (nmol)**	**cumulative amount / dose lungs**	**cumulative amount / air concentration**	**air conc.**	**total lung dose**	**available lung dose (26%)**	**cumulative amount metabolites (nmol)**	**cumulative amount / dose lungs**	**cumulative amount / air concentration**
		**nmol/ m³**	**nmol**	**nmol**	**Nmol**	**nmol / nmol dose**	**nmol / nmol/m³**	**nmol / m³**	**nmol**	**nmol**	**nmol**	**nmol / nmol dose**	**nmol / nmol/m³**
**1 (F)**	73	1158	1096	1096	1461	1.334	1.262	319	302	78.5	46.0	0.589	0.144
**2 (F)**	60	1158	900.4	900.4	904.2	1.004	0.781	319	248	64.6	79.0	1.229	0.247
**3 (F)**	73	1158	1096	1096	2722	2.485	2.351	319	302	78.5	353	5.388	1.105
**4 (F)**	61	1158	915.0	915.0	1274	1.392	1.100	319	252	65.6	23.0	0.346	0.072
**5 (F)**	64	1759	1460	1460	806.4	0.552	0.458	327	271	70.5	139	1.971	0.425
**6 (M)**	132	1759	3008	3008	250.8	0.083	0.143	327	559	145	8.0	0.058	0.024
**7 (F)**	82	1759	1868	1868	247.8	0.133	0.141	367	390	101	53.0	0.852	0.144
**8 (M)**	78	1759	1779	1779	1324	0.744	0.753	367	372	96.7	n.d.	n.d	n.d
**9 (M)**	85	1702	1874	1874	2602	1.388	1.529	223	246	63.9	86.0	0.830	0.386
**10 (F)**	85	n.d.	n.d	n.d	n.d	n.d.	n.d.	223	246	63.9	33.0	0.524	0.148
**11 (F)**	87	1702	1917	1917	854.6	0.446	0.502	223	251	65.3	28.0	0.427	0.126
**12 (M)**	68	1702	1501	1501	2316	1.543	1.361	223	197	51.1	45.0	0.873	0.202
**13 (M)**	82	1268	1347	1347	329.6	0.245	0.260	324	345	89.6	49.0	0.545	0.151
**14 (M)**	93	1268	1530	1530	n.d	n.d.	n.d.	324	391	102	105	1.031	0.324
**15 (F)**	74	1268	1217	1217	471.0	0.387	0.371	324	311	80.9	77.0	0.290	0.237
**16 (M)**	80	1268	1315	1315	70.05	0.053	0.055	367	381	99.1	96.0	0.968	0.261
**Mean**	**80**	**1456**	**1522**	**1522**	**1117**	**0.842**	**0.790**	**306**	**317**	**82.3**	**81.3**	**0.995**	**0.266**
**SD**	17	273.1	532.1	532.1	888.2	0.711	0.660	52.6	88.6	23.0	82.9	1.282	0.257
**CV(%)**	21	19	35	35	80	84	83	17	28	28	102	129	96

Table S4. Total excreted dose after combined inhalation and dermal and dermal exposure to D_4-DEP.
Excreted metabolite levels (in nmol) after combined, inhalation and dermal, exposure to D_4-DEP, as well as dermal exposure to D_4-DEP only. Values are given as cumulative amounts (nmol), and cumulative amounts normalized per total air concentration.

DEP		Inhalation and dermal				Dermal				Inhalation only ((inhalation and dermal) - (dermal))			
Subject (gender)	weight (kg)	air conc.	total lung dose	cumulative amount metabolites	cumulative amount / air concentration	air conc.	total lung dose	cumulative amount metabolites	cumulative amount / air concentration	air conc.	total lung dose	cumulative amount metabolites	cumulative amount / air concentration
		nmol/m³	nmol	nmol	nmol / nmol/m³	nmol/m³	nmol	nmol	nmol / nmol/m³	nmol/m³	nmol	Nmol	nmol / nmol/m³
**1 (F)**	73	1158	1096	1538	1.328	1224	-	76.7	0.063	1158	1096	1461	1.262
**2 (F)**	60	1158	900.4	998.4	0.862	1224	-	94.2	0.077	1158	900.4	904.2	0.781
**3 (F)**	73	1158	1096	2980	2.573	1224	-	258	0.211	1158	1096	2722	2.351
**4 (F)**	61	1158	915.0	1547	1.336	1224	-	274	0.223	1158	915.0	1274	1.100
**5 (F)**	64	1759	1460	1030	0.586	1242	-	224	0.180	1759	1460	806.4	0.458
**6 (M)**	132	1759	3008	272.3	0.155	1242	-	21.5	0.017	1759	3008	250.8	0.143
**7 (F)**	82	1759	1868	277.1	0.158	1242	-	29.3	0.024	1759	1868	247.8	0.141
**8 (M)**	78	1759	1779	1391	0.791	1242	-	67.5	0.054	1759	1779	1324	0.753
**9 (M)**	85	1702	1874	2766	1.626	1154	-	164	0.142	1702	1874	2602	1.529
**10 (F)**	85	n.a.	n.d	n.d	n.d	1154	-	62.6	0.054	n.a.	n.d	n.d	n.d
**11 (F)**	87	1702	1917	875.9	0.515	1154	-	21.2	0.018	1702	1917	854.6	0.502
**12 (M)**	68	1702	1501	2484	1.460	1154	-	169	0.146	1702	1501	2316	1.361
**13 (M)**	82	1268	1347	450.9	0.355	1154	-	121	0.074	1268	1347	329.6	0.260
**14 (M)**	93	1268	1530	1295	1.021	n.d.	-	n.d.	n.d.	1268	1530	n.d	n.d
**15 (F)**	74	1268	1217	600.2	0.473	1631	-	129	0.079	1268	1217	471.0	0.371
**16 (M)**	80	1268	1315	139.4	0.110	1631	-	69.3	0.043	1268	1315	70.05	0.055
**Mean**	**80**	**1456**	**1522**	**1243**	**0.890**	**1260**		**119**	**0.094**	**1456**	**1522**	**1117**	**0.790**
**SD**	17	273.1	532.1	904.6	0.681	155.4		83.0	0.069	273.1	532.1	888.2	0.660
**CV(%)**	21	19	35	73	77	12		70	74	19	35	80	83

Table S5. Analytical details for quantification of phthalate esters using GC-MS/MS.
Retention time (RT), quantitative and qualitative ion transition, collision energies (CE), limit of detection (LOD) and limit of quantification (LOQ), as well as quality controls (QC) at 200 ng/mL (n = 10), 500 ng/mL (n = 10), 1000 ng/mL (n = 10) and coefficient of variation (CV).

**Compound**	**RT**	**Transition Quant**	**CE (eV)**	**Transition Qual**	**CE (eV)**	**LOD (ng/mL [pmol/mL]**	**LOQ ng/mL [pmol/mL]**	**QC 200 ng/mL**	**QC 500 ng/mL**	**QC 1000 ng/mL**
**D_4-DEP**	5.24	153 -> 69	30	153 -> 97	20	1.0 [4.4]	3.4 [15.0]	295 (CV 9.5%)	486 (CV 5.8%)	1021 (CV 4.7%)
**D_4-DEHP**	9.89	153 -> 69	25	153 -> 97	20	4.7 [11.9]	15.8 [40.0]	268 (CV 5.8%)	497 (CV 7.0%)	1005 (CV 3.3%)
**$^{13}C_6$-D_4-DEP**	5.22	159 -> 74	25							
**$^{13}C_6$-D_4-DEHP**	9.87	159 -> 74	20							

Table S6. Analytical details for quantification of phthalate metabolites using LC-MS/MS.
Retention time (RT), quantitative MRM transitions, collision energies (CE), limit of detection (LOD) and limit of quantification (LOQ); quality controls at 8 ng/mL (*n* = 10) and 80 ng/mL (*n* = 10) and coefficient of variation (CV).

Compound	RT	Transition	CE (eV)	Internal standards	RT	Transition	CE (eV)	LOD ng/mL [pmol/mL]	LOQ ng/mL [pmol/mL]	QC 8 ng/mL	QC 80 ng/mL
**D_4-MEP**	4.56	197.1-> 81.0	22	**D_9-MEP**	4.18	202.1 -> 86.0	22	0.5 [2.5]	1.7 [8.5]	9.6 (CV 36%)	82.1 (CV 22%)
**D_4-MEHP**	5.61	281.3-> 136.8	22	**D_4-9cx-MEPP**	4.74	316.1 -> 125.0	34	0.9 [3.3]	2.8 [9.9]	7.1 (CV 51%)	55.2 (CV 56%)
**D_4-5OH-MEHP**	4.89	297.1-> 125.0	24	**D_4-9cx-MEPP**	4.74	316.1 -> 125.0	34	0.1 [0.3]	0.5 [1.7]	6.6 (CV 47%)	63.5 (CV 57%)
**D_4-5oxo-MEHP**	4.93	295.1-> 125.0	24	**D_4-9cx-MEPP**	4.74	316.1 -> 125.0	34	0.2 [0.6]	0.6 [2.0]	9.8 (CV 56%)	78.8 (CV 53%)
**D_4-5cx-MEPP**	4.76	311.2 -> 159.2	19	**D_4-9cx-MEPP**	4.74	316.1 -> 125.0	34	0.1 [0.3]	0.4 [1.2]	9.8 (CV 47%)	79.2 (CV 58%)
**D_4-2cx-MEPP**	4.78	311.2 -> 159.2	19	**D_4-9cx-MEPP**	4.74	316.1 -> 125.0	34	0.1 [0.3]	0.5 [1.6]	5.3 (CV 55%)	84.8 (CV 72%)

GC-MS/MS—Method details

Suppl. Table 5 shows the quantifier ions and qualifier ions, retention times and collision energies for D_4-DEP and D_4-DEHP and internal standards. All runs included at least 10 blank samples and three unused tenax tubes were extracted and analyzed for background contamination that was subtracted from all values. All samples from one individual exposure scenario were analyzed in the same run. The specificity was tested by analyzing extracted blank tenax tubes spiked with internal standard. Excellent linearity was seen for the calibration standards ranging from 50 to 1000 ng/mL in toluene. The correlation coefficient (r^2) observed for D_4-DEP was above 0.983 and D_4-DEHP was above 0.973. LOD and LOQ were determined by analysis of 10 different blank toluene samples spiked with internal standard. The LOD was calculated as three times the standard deviation of the ratio between the peak area at the analyte retention time and the peak area of internal standard, divided by the slope of the calibration line. The LOQ was similarly determined as 9 times the standard deviation of the ratios. The determined LOD ranged from 1.2 ng/mL (D_4-DEP) to 5.5 (D_4-DEHP) ng/mL, and LOQ values were in between 4.0 (D_4-DEP) and 18.3 ng/mL (D_4-DEHP) (Table S5). The within-run precision of the method was determined by analyses of 10 toluene samples spiked with 200, 500 and 1000 ng/mL of D_4-DEHP or D_4-DEP, respectively. The CV at the three concentration levels of the analyzed compounds was below 10% (Table S5). The between-run precision was determined by incorporating three QC samples in duplicates in every analytical batch. The mean quantified values at the three concentration levels (200, 500, 1000 ng/mL) were 268, 497 and 1005 ng/mL for D_4-DEHP and 295, 486, and 1021 ng/mL for D_4-DEP (Table S5).

LC-MS/MS—Method details

Suppl. Table 6 presents the ion transitions for all quantified phthalate metabolites and internal standards, as well as collision energies and retention times. All runs included at least 10 blank samples, and background contamination was subtracted from all values. Excellent linearity was seen for the calibration standards ranging from 0 to 1000 ng/mL in acetonitrile/water (50:50). The correlation coefficient (r^2) observed was above 0.990 (D_4-MEP), 0.993 (D_4-MEHP), 0.980 (D_4-5OH-MEHP, D_4-5oxo-MEHP, D_4-5cxMEPP) and 0.900 (D_4-2-cxMMHP). LOD and LOQ were determined by analysis of 10 different blank toluene samples spiked with internal standard. The LOD was calculated as three times the standard deviation of the ratio between the peak area at the analyte retention time and the peak area of internal standard, divided by the slope of the calibration line. The LOQ was similarly determined as 10 times the standard deviation of the ratios. The determined LOD for all analysed metabolites were: 0.5 ng/mL (D_4-MEP), 0.9 ng/mL (D_4-MEHP), 0.1 ng/mL (D_4-5OH-MEHP), 0.2 ng/mL (D_4-5oxo-MEHP), 0.1 ng/mL (D_4-5cx-MEPP), 0.1 ng/mL (D_4-2cx-MMHP) (Table S6). The within-run precision of the method was determined by replicate analyses of 10 urine samples spiked with 8 and 80 ng/mL of D_4-labelled metabolite standards, with a CV below 10% (Table S6). The between-run precision was determined by incorporating two QC samples in duplicates in every analytical batch. The mean quantified values at the two concentration levels (8 and 80 ng/mL) were 82.1 ng/mL (D_4-MEP), 67.4 ng/mL (D_4-MEHP), 73.9 ng/mL (D_4-5OH-MEPP), 73.2 ng/mL (D_4-5oxo-MEHP), 74.8 ng/mL (D_4-5cx-MEPP) and 71.1 ng/mL (D_4-2cx-MMHP) (Table S6).
